# Exploring user engagement with real-time verbal feedback from an exoskeleton-based virtual exercise coach

**DOI:** 10.1177/20552076241302652

**Published:** 2024-12-05

**Authors:** Raju Maharjan, Sanjana Mendu, Milton Mariani, Saeed Abdullah, John Paulin Hansen

**Affiliations:** 1 6187 The University of Oklahoma - Norman Campus, Norman, USA; 2 Department of Informatics, 8082 Pennsylvania State University, University Park, PA, USA; 3 5205 Technical University of Denmark, Lyngby, Hovedstaden Denmark

**Keywords:** Conversational agents, virtual exercise coach, speech recognition, natural language interface, real-time feedback, engagement, user experience, exoskeleton

## Abstract

**Objective:**

Engaging users during physical exercise is crucial for fostering long-term commitment, however, sustaining that engagement remains a significant challenge. This study explores the design of a voice-enabled exoskeleton-based virtual exercise coach (VEC) that provides real-time verbal feedback to enhance user engagement. The objectives of this study are twofold: (i) to compare user engagement with real-time verbal feedback from both VEC and human exercise coach (HEC) during physical exercise, and (ii) to understand users’ perceptions and gather their recommendations for improving future VEC technologies.

**Methods:**

We developed an exoskeleton-based VEC that delivers real-time verbal feedback on users’ exercise performance. To evaluate its impact on user engagement, we conducted a lab-based mixed-methods study ( N=32 ) over a period of 6 weeks comparing users’ engagement with the VEC and HEC using User Engagement Scale (UES) questionnaire and conducted semi-structured interviews to understand users’ perceptions of the VEC.

**Results:**

Participants in this study found the VEC more engaging than the HEC, in terms of focused attention ( Z=156.5,p<.001 ) and perceived usability ( Z=32,p<.001 ). Post-interaction interviews revealed that (i) users found the VEC to be engaging, intuitive, easy to use, and convenient; (ii) users perceived the VEC as a valuable training companion that could help reduce the emotional insecurities often associated with going to the gym; and (iii) users expressed a desire for the VEC to have a personality and embodiment that motivates and supports personalized interactions.

**Conclusion:**

Based on our results, we discuss the challenges and implications for designing future voice-enabled VECs that support engaging physical exercises.

## Introduction

Regular physical exercise has well-established health benefits, including a lower risk of cardiovascular disease, diabetes, and hypertension. It also has positive effects on mental health, such as delaying the onset of dementia and reducing symptoms of depression and anxiety.^1,2^ The 2018 Physical Activity Guidelines for Americans (PAGA18) recommend that adults engage in at least 150–300 min per week of moderate physical activity, 75–150 min per week of vigorous physical activity, or an equivalent combination of both intensities in addition to muscle-strengthening activities 2 or more days per week.^3^ Despite the benefits and efforts to promote regular exercise, over a quarter of the world’s adult population (1.4 billion) remains insufficiently active, undermining not only their physical and mental health but also straining healthcare systems globally.^4,5^ This highlights a pressing need for more effective strategies to engage people in regular exercise.

### Virtual exercise coaches (VECs) to support exercise engagement

Physical exercise engagement is affected by various factors, including environmental, socio-cultural, psychological, and physical aspects.^6–8^ One important aspect contributing to exercise engagement is the way individuals feel and their level of enjoyment during physical activity.^9^ Studies indicate that novel technologies and interactive experiences have the potential to positively impact users’ exercise behavior, thereby increasing exercise rates and overall physical activity.^10,11^

VECs have emerged as promising way to support sustained engagement with physical exercise. A VEC can be defined as a system that supports individuals during their exercise routines by providing verbal and/or visual feedback on their performance, goal and providing encouragement and guidance, aiming to replicate the role of a human fitness coach.^12,13^ These systems have been implemented using a wide array of technological platforms, including virtual reality (VR),^14–17^ embodied conversational agents (ECAs),^18,19^ social robots,^20–22^ and wireless sensors.^23,24^ Prior work has established the efficacy of VECs in promoting increased frequency and intensity of physical exercise across a wide array of populations and contexts.^17,15,25,26^ However, promoting active user engagement with these systems, towards positively influencing exercise behavior and increasing overall physical activity levels, poses a significant challenge.

Prior research on VEC has measured behavioral engagement as frequency or duration of system use^27–29^ on the assumption that usage is closely related to outcome. Bort-Roig et al.^30^ found that the majority of recent studies which used smartphone technology to support engagement with physical activity measured engagement via frequency of app use. Mouatt et al.^15^ also found that measures of engagement with VECs largely focused on behavioral outcomes, such as short-term attendance rates. Psycho-physiological measurements, ranging from energy expenditure and heart rate to facial expression and gaze detection, have also been used to measure users’ cognitive engagement with VECs.^15,31,32^

While these aspects of engagement provide valuable context for supporting meaningful and sustained user interactions with VECs, recent work has highlighted the limitations of such conceptualizations. For example, Yardley et al.^33^ argued that focusing on effective rather than sustained engagement is critical to supporting positive health behavior change via digital intervention technologies. While behavioral and cognitive approaches to measuring engagement allow researchers to capture objective measurements of users’ interactions with VECs, there is a need to move beyond these dimensions toward more holistic evaluations of user experience. To this end, Doherty and Doherty^34^ highlighted the utility of including considering users’ emotional engagement with novel technological systems. Emotional aspects of engagement, such as the way individuals feel and their level of enjoyment, have been shown to significantly impact people’s experience of physical activity.^9^ For example, how people feel during exercise has been shown to predict their future exercise engagement,^35^ suggesting that enhancing individuals’ experiences during exercise may have important influences on their future exercise behavior.

In addition to the gap between investigations of behavioral vs. emotional engagement, there is a notable lack of studies examining in-the-moment engagement with VECs. Yardley et al.^33^ highlighted the important distinction between micro-level (i.e. moment-to-moment) and macro-level (i.e. long-term behavior change) engagement and argue that, while they are closely connected, a detailed understanding of both the features that influence use of the technology and motivate changes in behavior is important to informing the design of effective interventions. Oertel et al.^36^ re-iterated this distinction and highlight the complex interplay between short-term and long-term engagement, further pointing to the need to address this gap.

Toward capturing in-the-moment engagement with VECs, recent studies have measured users’ attentional focus during interactions using methods such as facial expression analysis^32^ and gaze tracking.^22^ However, these measures overlook emotional aspects of engagement and limited research has studied experiential aspects of real-time verbal feedback. The present work aims to address this gap by investigating both behavioral and emotional dimensions of in-the-moment engagement with a VEC.

### VEC real-time verbal feedback design

Research suggests that providing real-time feedback to users about the way they exercise can lead to improvements in performance^37,38^ and attitude toward exercise.^39^ Wilson et al.^40^ found that providing real-time quantitative performance feedback can improve motivation, competitiveness, mood, and exercise performance. A recent meta-review by Braakhuis et al.^41^ on the effect of providing objective feedback on physical activity performance found a moderately positive effect of feedback (as compared with no feedback) on desired health outcomes. Verbal feedback in particular has been shown to have positive effect in physical performance, psychophysiological responses, and motivation during physical exercise.^12,37,38,42,43^

Prior work on supporting exercise via VECs has focused heavily on providing visual feedback. A recent review conducted by Monteiro-Guerra et al.^44^ found that most existing VECs provide some form of visual feedback, either through graphs^23,28,39,45^ or more complex visual displays such as avatars^46^ or virtual ecosystems that change based on the user state.^16^ Studies that used a social robot as a VEC made use of the robot’s tangible form by having it demonstrate the exercise to the user.^21,32,47,48^

Despite promising findings from these studies, there is evidence to suggest that visual modalities are insufficient for effective delivery of feedback. For example, Kim and Kramer^49^ found that visual feedback had diminishing effects on exercise performance over time, suggesting that visual feedback is most helpful for learning and may be less important for the performance of well-learned tasks. This has serious implications for the efficacy of VECs in supporting vulnerable populations (e.g. patients in clinical settings) who would benefit most from longitudinal use of the system. Furthermore, Hermsen et al.^50^ argue that while visual feedback may be more effective in communicating more detailed information, verbal delivery of feedback could be less disruptive while the recipient is engaged in an activity. Together, these advantages suggest that moving away from visual and toward verbal feedback could be uniquely beneficial in the context of VECs and exercise engagement.

Although some recent work has begun to investigate the advantages of verbal feedback from VECs in supporting exercise engagement, prior studies have primarily focused on the use of simple auditory signals. For example, Mazilu et al.^51^ developed a system that uses a rhythmic auditory signal to alert the user to potential freeze of gait episodes. Monteiro-Guerra et al.^44^ identified three VEC systems which explored the use of verbal feedback, all of which consisted of a buzzing or musical tone. While these auditory feedback are useful as tools for one-way signals of information, more advanced verbal interactions (e.g. natural language) have the potential to more interactively change users’ behavior and attitudes.^52^ Using natural language to interact with users, a VEC could not only create an environment in which users experience exercise in a more exciting and motivating way but also become a personal trainer, taking on individualized tones of voice and turns of phrase that best suit the user and their dynamic contexts.^53^ However, prior work has largely measured user experience with these systems in terms of behavioral outcomes (i.e. increased physical activity). As a result, our understanding of how the use of real-time feedback impacts in-the-moment user engagement and their perceptions of the system remains limited.

### Current study

This study explores the following three research questions to understand how users engage with VEC’s real-time verbal feedback on their exercise performance and their overall perception of the technology, with an aim to guide the future design of voice-enabled VEC:

RQ1:  How does user engagement with a voice-enabled VEC compare to a human exercise coach (HEC)?
RQ2:  How do users perceive engaging with a voice-enabled VEC for physical exercise?
RQ3:  What are the challenges of designing a voice-enabled VEC, and what are their implications for future VEC design?

By comparing user engagement between VEC and HEC (RQ1), we seek to gain insights for designing more engaging VEC that can potentially enhance user experience and fitness outcomes. The comparison may also highlight the strengths and limitations of each approach, guiding future technology development. Additionally, understanding users’ perceptions of VEC (RQ2) can help designers tailor the technology to users’ needs and preferences, leading to a more engaging and effective exercise experience, which can contribute to improved user adherence and fitness outcomes. Finally, knowledge of potential challenges in designing a VEC (RQ3) can help designers to proactively address these issues, leading to improved future VEC designs.

Using a lab-based mixed method approach, the current study makes the following contributions: (i) the finding that the participants in this study rated their in-the-moment engagement with the VEC significantly higher compared to HEC, in terms of focused attention and perceived usability as measured by the User Engagement Scale (UES) questionnaire; (ii) insights into users’ perception of the VEC technology as a valuable training companion that can help reduce the emotional insecurities often associated with going to the gym, (iii) recommendations for enhancing user engagement by giving the VEC a personality, creating a sense of presence through embodiment, integrating more motivational cues, and supporting personalization, and (iv) implications for future VEC designs based on challenges identified in this study, such as addressing uncanny VEC-user interactions, integrating social dynamics and presence, and improving VEC responsiveness and personalization.

## Method

We developed a voice-enabled exoskeleton-based VEC that provides real-time verbal feedback on users’ exercise performance. We conducted a lab-based mixed-methods^54–56^ study over a period of 6 weeks. The objectives of this study were (i) to conduct a comparative analysis of users’ engagement with real-time verbal feedback from both VEC and HEC during physical exercise, and (ii) to gain an understanding of users’ experiences and perceptions of the VEC, with the aim of informing the future design of similar technologies.

### VEC implementation

**Figure 1.**
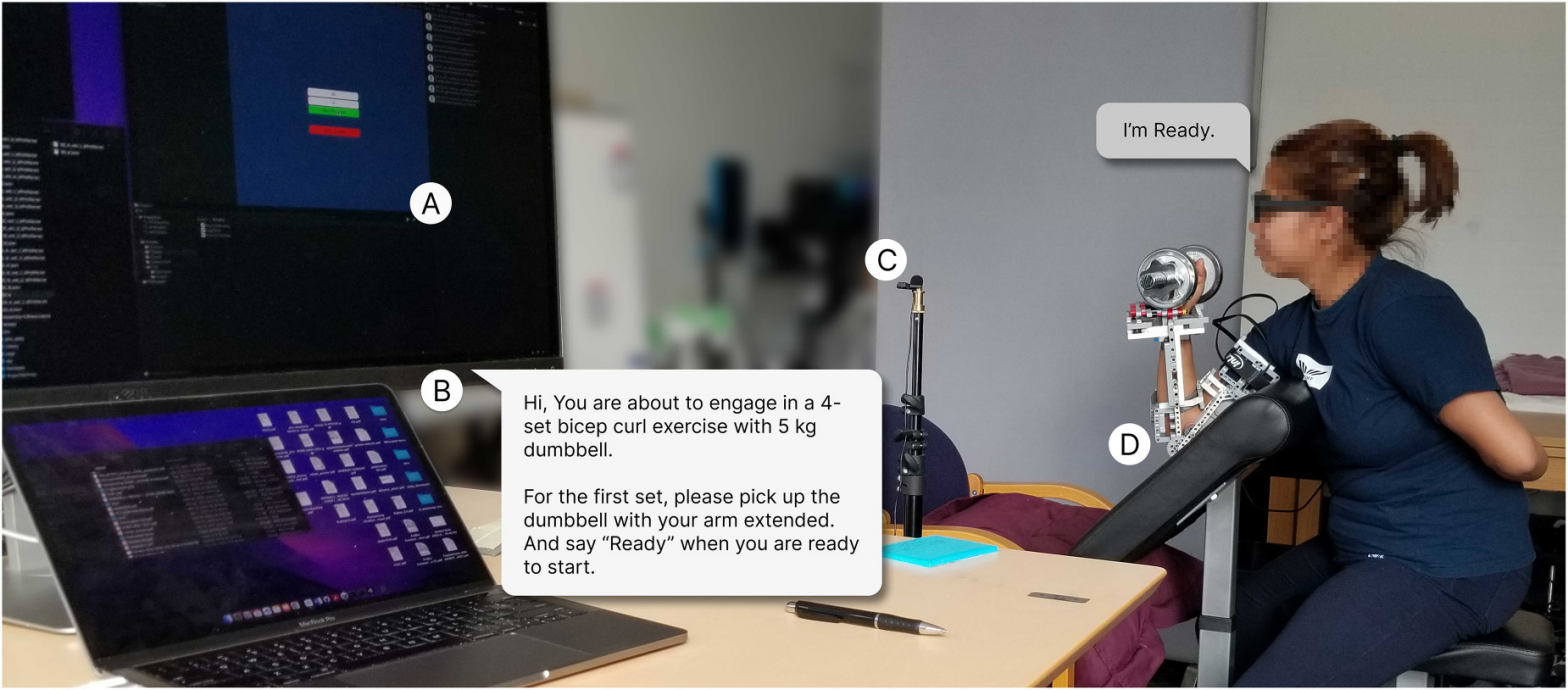
Components of the VEC. A: GUI comprising input fields to enter participant ID and dumbbell weight, B: inbuilt speaker in the monitor for the VEC’s verbal output and a speech bubble demonstrating a sample instruction from the VEC, C: microphone connected to the laptop to capture’s users’ responses, and D: Lego exoskeleton with motor sensors. VEC: virtual exercise coach; GUI: graphical user interface.

In order to allow participants to receive real-time verbal feedback on their physical exercise through a VEC, we modified an exoskeleton-based rehabilitation system for elbow flexion and extension originally developed by Homola et al.^57^ As shown in Figure 1, the system featured speech interaction using Microsoft Cognitive Services, allowing users to verbally interact with the system and receive diverse types of feedback on their performance of a one-arm dumbbell bicep curl exercise. Additionally, the system included a graphical user interface (GUI) that enabled researchers to manually start and stop exercise sessions, as well as collect data on the weight of the dumbbell and participant ID.

The VEC prototype was constructed out of Lego Technic Bricks to allow for easy and rapid modification, testing, and revision. The exoskeleton’s motors acted as sensors and provided motor angle position representing arm position, used to provide rule-based verbal feedback on the users’ performance.

We adapted verbal feedback methods from previous research (e.g. Pacholek and Zemková,^12^ and Kowatsch et al.^13^) and consulted with a professional trainer to integrate them into our system. The different feedback types reflect established behavior change techniques (BCT) for increasing physical activity.^58^ Specifically, we prompt rewards contingent on effort or progress towards behavior (verbal encouragement; BCT 12), provide feedback on performance (performance feedback; BCT 19), provide instruction on how to perform the behavior (guidance; BCT 21), and prompt practice (goal-oriented feedback; BCT 26). As shown in Table 1, VEC introduces the exercise to the participant before the start of the session (State 1). VEC prompts the dumbbell weight, number of sets and repetitions, instructs to pick up the weight, and asks to confirm if the user is ready to start. Upon user’s confirmation (State 2), it instructs the user to perform the exercise by voicing “let’s go.” and starts counting the repetitions (State 3). VEC then guides the user by providing real-time verbal feedback on the count of the repetitions (State 4).

**Table 1.** Real-time verbal feedback on the proposed VEC.

State	Feedback type	Purpose	Trigger	Example
(1) Introduction	GF	Inform user about the session goal	Session start	VEC: Hi, You are about to engage in a 4-set bicep curl exercise with {weight} kg dumbbell.
(2) Confirmation	C	Get user confirmation	Introduction is complete or the 10-second break between sets is complete	For the {set number} set, please pick up the {weight} kg dumbbell with your arm extended. And say “Ready” when you are ready to start.
(3) Session start	G	Provide training instructions	User confirmed by saying “Ready”	Alright, Let’s go!
(4) Repetition Counting	PF	Count repetitions		1, 2, 3, 4,…
(5) Task encouragement	GF	Inform user to lift up the dumbbell to complete the repetition	Elbow flexion decreases by >5° before reaching upper border	Up up up!
(6) General encouragement	VE	Motivate user to complete the repetition	Repetition length >3 seconds	C’mon you can do it!
(7) Set completion	PF	Praise user for completing a set	After praising the user for completing a set	“Clapping sound.” That was awesome! You completed the {number} set.
(8) Start break	G	Provide training instructions	User completed a set (10 repetitions)	Lets take a 10 second break.
(9) Session end	C	Indicate end of the session	User completed all four sets or ended the session manually	Thank you for participating in the study. Bye!

**C:** confirmation; **G:** guidance; **GF:** goal-oriented feedback; **PF:** performance feedback; **VE:** verbal encouragement.

During each session, VEC calculates the user’s elbow flexion from their resting position to the upper border when lifting the dumbbell, and then back to the resting position, which counts as one repetition. The value for the resting position in each session is specified based on the motor angle of the user’s elbow extension position. The upper border is set at 90° plus the value of the user’s resting position.

If the user has difficulty completing the repetition, as indicated by a drop of at least five degrees before reaching the upper border during elbow flexion, the system prompts them to lift the dumbbell by voicing “Up up up!” (State 5). Similarly, if the user takes more than three seconds to complete a repetition, the system provides encouragement with the phrase “C’mon you can do it” (State 6). We determined these thresholds for triggering feedback through trial-and-error approach involving five individuals. In the case when a participant is unable to complete the set, they can ask the researcher to quit the program. After completion of each set, the system praises the users on their performance (e.g. clapping sound and “Well done!,” or “Awesome!” or “That was great!”) (State 7) and asks the user to take a 10 second break (State 8) before starting the next set (State 2). At the end of the last set in a session, VEC thanks the user for completing the exercise and participating in the study in addition to praising the user (State 9).

In the case of “no match” errors (when the system fails to understand the response) during the session, the system provides fallback prompts (e.g. “Sorry, I didn’t get that. Please say ‘Ready’ when you are ready to start.”). Respondents have three attempts to respond to the system’s prompts. After three re-prompts, VEC ends the session requiring to start a new session.

### Participants

Thirty two participants ( n=32 , F = 14 ) with mean age of 27.5 were recruited from the Technical University of Denmark via internal email, posters, and word of mouth. Table 2 provides an overview of participants’ demographic characteristics. 75 % ( n=24 ) of participants reported that they had prior experience using virtual agents like Alexa, Google assistant or Siri and 46 % ( n=15 ) reported having prior experience with human coach for exercises. While the majority of participants ( 90.63% ; n=29 ) identified as right handed, 3 participants ( 9.38% ) identified as left handed.

**Table 2.** Participant demographics.

ID	Sex	Age	Education	Technical ability	VA Exp.	Coach Exp.	Handedness	TTM
1	M	24	Upper Sec	Good	No	No	Right	Action
2	M	25	Ms/PhD	V. good	Yes	No	Right	Maintenance
3	M	25	Bs	V. good	Yes	Yes	Right	Maintenance
4	M	24	Ms/PhD	V. good	Yes	Yes	Left	Preparation
5	F	21	Below Deg.	Fair	No	No	Right	Action
6	F	21	Bs	V. good	Yes	Yes	Right	Contemplation
7	F	27	Bs	Good	No	No	Right	Maintenance
8	M	26	Ms/PhD	V. good	Yes	Yes	Right	Action
9	M	21	Bs	V. good	Yes	No	Right	Maintenance
10	M	23	Bs	V. good	No	No	Left	Action
11	M	24	Ms/PhD	V. good	Yes	No	Right	Maintenance
12	M	30	Ms/PhD	V. good	Yes	Yes	Right	Maintenance
13	F	47	Ms/PhD	V. good	No	Yes	Right	Action
14	M	39	Ms/PhD	V. good	Yes	No	Right	Maintenance
15	F	24	Ms/PhD	V. good	Yes	No	Left	Maintenance
16	F	23	Ms/PhD	V. good	Yes	No	Right	Action
17	F	24	Ms/PhD	Good	No	Yes	Right	Maintenance
18	F	25	Bs	V. good	Yes	Yes	Right	Maintenance
19	F	21	Bs	V. good	Yes	No	Right	Maintenance
20	M	21	Bs	Good	Yes	Yes	Right	Maintenance
21	M	23	Bs	V. good	Yes	No	Right	Maintenance
22	F	22	Bs	Fair	Yes	Yes	Left	Maintenance
23	M	21	Bs	V. good	Yes	No	Right	Maintenance
24	M	31	Ms/PhD	V. good	Yes	Yes	Right	Maintenance
25	M	40	Ms/PhD	Good	No	Yes	Right	Maintenance
26	F	38	Bs	Good	Yes	Yes	Right	Maintenance
27	M	38	Ms/PhD	V. good	No	No	Right	Maintenance
28	M	39	Ms/PhD	Good	Yes	No	Right	Preparation
29	F	33	Bs	Good	Yes	Yes	Right	Action
30	M	26	Bs	V. good	Yes	No	Right	Pre-contemplation
31	F	23	Ms/PhD	V. good	Yes	Yes	Right	Maintenance
32	F	30	Bs	Good	Yes	No	Right	Maintenance

VA: virtual agent; exp.: prior experience with VA.

With respect to behavioral intention, 65% ( n=21 ) of participants identified themselves as being in the maintenance stage, while 21% ( n=7 ) reported being in the action stage according to the Stage of Change for Physical Activity questionnaire.^59^ This questionnaire assesses individuals’ position within the five stages of the Transtheoretical Model of Behavioral Change (TTM),^60^ which includes precontemplation, contemplation, preparation, action, and maintenance. The precontemplation stage describes a lack of intention to engage in behavior change in the near future (i.e. next 6 months). The contemplation stage describes intention and concrete plans to engage in behavior change in the near future (i.e. the next 6 months). The preparation stage describes intention and preliminary actions to engage in behavior change. The action stage describes engagement with behavior change for a short period of time. Finally, the maintenance stage describes successful behavior change and maintenance over a long period of time.

### Study design and procedure

**Figure 2.**
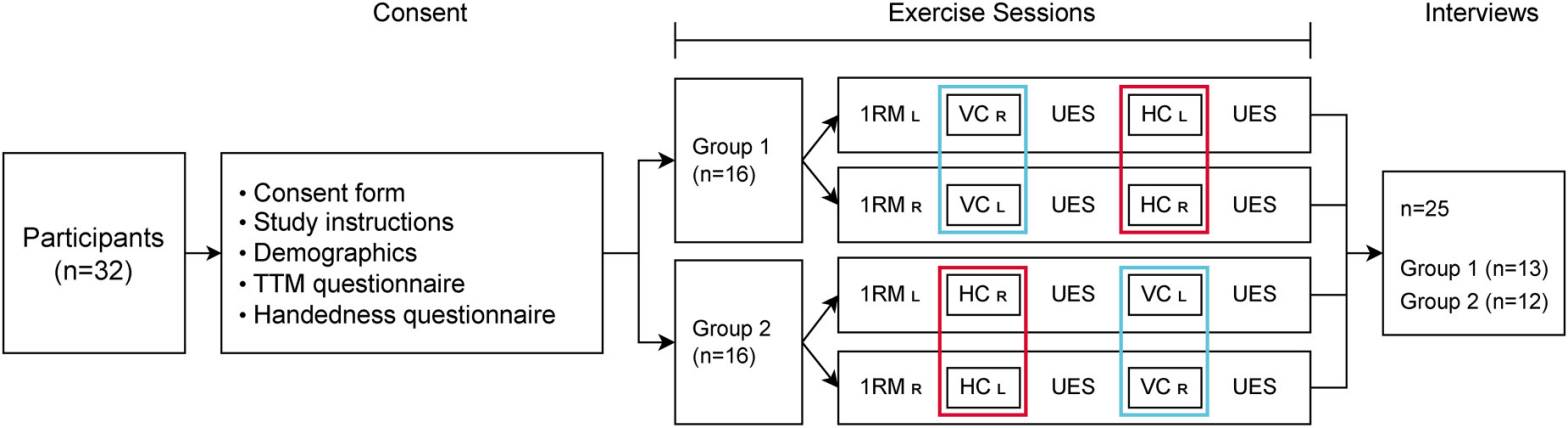
Experimental design and procedure. RM: 1 Rep Max; UES: User Engagement Scale; L: left hand; R: right hand.

We conducted a lab-based within-group experiment counterbalanced by a 2× Latin square design to compare users’ engagement with real-time verbal feedback from both VEC and HEC during physical exercise (see Figure 2). Participants were randomly assigned to one of following two versions of the system, each delivering identical verbal feedback, as outlined in Table 1:

- *VEC*. In this version of the system, users interacted with the virtual coach with no supervision and feedback by a human coach.
- *HEC*. This version of the system involved a human coach who visually monitored user’s performance, and provided feedback based on their observations. To alleviate potential confounding variables such as the human coach’s non-verbal cues, the coach stood behind the participant’s side during the study session and adhered to the same feedback rules as the VEC.

#### Participant consent

This study was exempt from ethical approval by the Danish National Committee on Health Research Ethics in accordance with Section 14 (2) of the Danish Act on Research Ethics Review of Health Research Projects.^61^ Prior to the study, participants were informed about the aim of the study and written consent from each participant was obtained. Additionally, demographic information was collected, including age, sex, education level, self-assessment of technical ability, prior experience using VA (e.g. Alexa, Google Nest) and human coach. They were also asked to fill out the Edinburgh Handedness Inventory questionnaire^62^ to determine the their dominant hand.

Participants were then demonstrated on how to interact with the system and informed about its capabilities and limitations. For example, the system counts each repetition of the bicep curl as a full cycle of elbow flexion and extension, the motor in the system does not function as an actuator during the exercise, and if the participant is unable to complete a set and wants to stop in the middle of the session, they may do so by informing the researcher.

#### Exercise sessions

As shown in Figure 2, participants were randomly assigned to either Group 1 or Group 2. Group 1 involved engaging in the bicep curl exercise session with the VEC followed by HEC, while Group 2 involved engaging with HEC first, followed by the VEC. Before engaging in the exercise session, each participant’s physical strength level for the bicep curl exercise, also called the one-rep max (1RM), was estimated. This process involved each participant selecting the maximum weight they could lift for five repetitions of the bicep curl exercise in a sitting position on a preacher bench. Once the participant confirmed their self-selected maximum weight for five repetitions (5RM), we used the equation ( 1RM=1.1307*5RM+0.6998 ) proposed by Reynold et al.^63^ to calculate their 1RM.

Adopting a protocol from a prior study by Mangine et al.^64^ [Table 1], the participants performed a four-set bicep curl exercise during every session. Each set consisted of ten repetitions using a dumbbell weight that was 70% of their 1RM (one-rep maximum) and a 10 second break between sets. In order to mitigate the potential influence of hand dominance as a confounding factor, participants alternated hands to calculate 1RM and to engage with the two conditions of the system. The exercise sessions were audio recorded.

After each exercise session, participants filled out the short form of the UES questionnaire,^65^ which is a validated 12-item, 5-point Likert scale. We chose this questionnaire because it provides a comprehensive measure of user engagement across multiple dimensions namely, focused attention, perceived usability, esthetic appeal and reward. These dimensions capture functional, emotional and cognitive aspects of the user experience, allowing for a nuanced comparison of user engagement between VEC and HEC. Such detailed insights are essential for designing an engaging system.

Data collected through the system included participants’ start and end timestamp of each repetition in a set, number of errors in user-VEC interaction, and the number of times a participant was provided motivational feedback.

#### Interviews

A total of 25 out of the 32 participants participated in one-on-one interviews with a member of the research team after completing the exercise session. The remaining seven participants opted out due to personal time constraints. During the interviews, participants shared their experiences with the system, expressed their overall opinions on the technology, and provided insights on the future design of VEC. Interviews lasted from 12 to 30 minutes and were audio recorded using Otter.^a^

### Analysis

We used a mixed method approach to analyze the data collected as part of this study. Our quantitative analysis centered around participants’ responses to the UES questionnaire, which characterizes user engagement with novel technological systems. Our qualitative analysis focused on the post-session interviews in which participants shared open-ended feedback regarding their experience with the VEC.

#### Quantitative

Participants’ responses to the UES questionnaire were analyzed using R (v. 4.2.1). We assessed the normality of the data by examining the distribution of residuals for each dimension of the questionnaire. In all cases, the residuals did not conform to a normal distribution. Therefore, we employed non-parametric Wilcoxon test^66^ statistics to compare participants’ subjective assessments of their engagement with both conditions of the system. Specifically, we compared participants’ self-reported focused attention, perceived usability, esthetic appeal, and reward for the HEC versus VEC.

#### Qualitative

We used Braun & Clarke’s inductive thematic analysis approach^67,68^ to qualitatively analyze the post-session interview data. We chose an inductive analysis approach to allow for flexibility in uncovering broader insights and generate recommendations for future VEC design, which aligns well with the exploratory nature of this study. Authors 1 and 3 began by reviewing the interview transcripts together and generating initial codes. Data saturation was confirmed when existing codes repeatedly appeared across participants’ transcripts and no new codes were found in later coding iterations. These codes were then grouped, along with supporting quotes, into categories to form candidate themes. These candidate themes were iteratively reviewed and refined with the input from author 2, leading to the final three themes presented in the results section. During the process, both semantic and latent levels of meaning in the data were considered, towards capturing a comprehensive view of participants’ experiences with the VEC and their perceptions of this technology.

## Results

**Figure 3.**
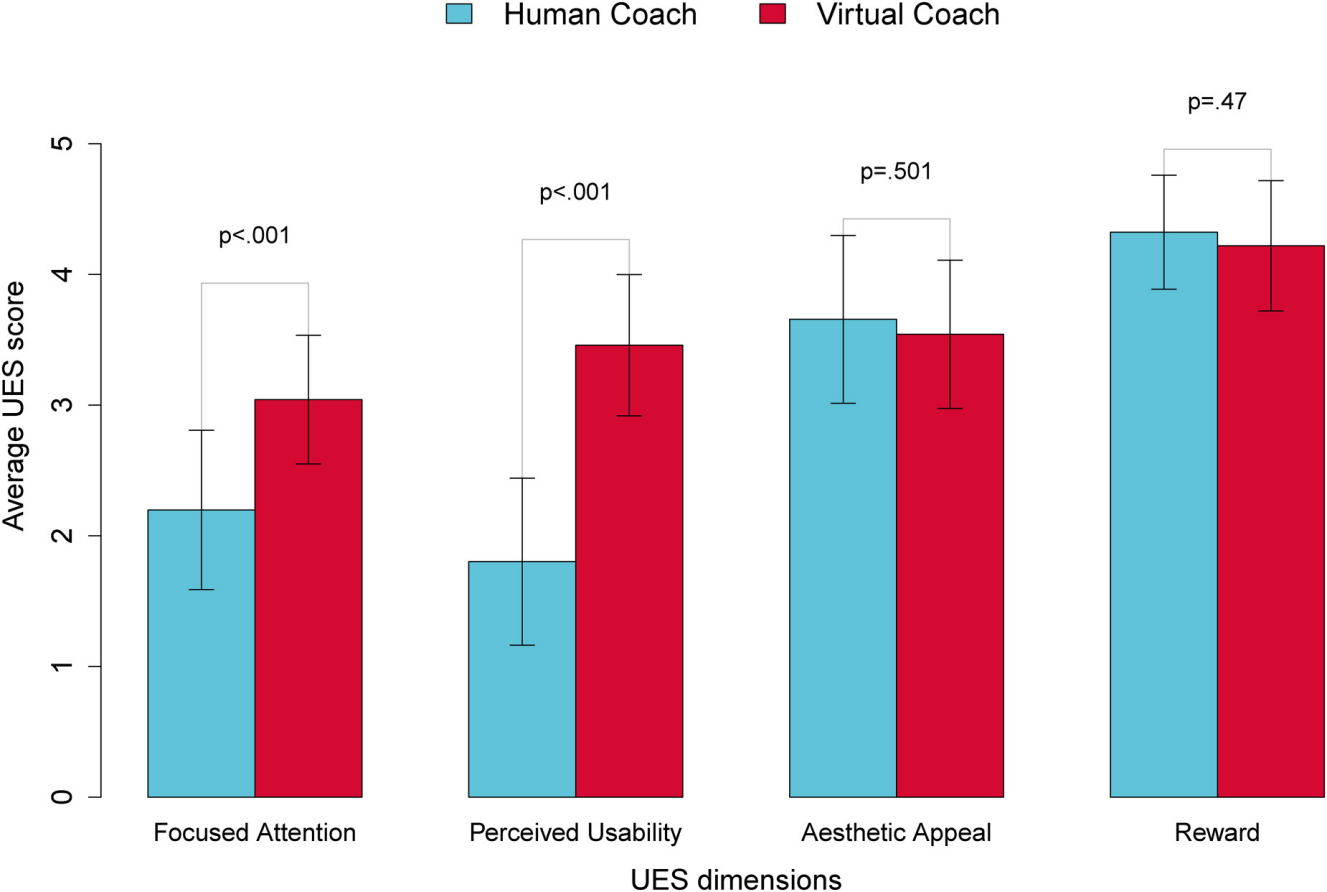
Combined bar-chart contrasts participants’ responses to the UES questionnaire, comparing their engagement levels between VEC and HEC on a scale of 1 to 5. Error bars reflect standard error and mean scores across all four dimensions. Note that the directionality of these constructs is based on the instructions provided alongside the scale (i.e. the higher the score, the better the engagement). UES: User Engagement Scale; VEC: virtual exercise coach; HEC: human exercise coach.

As shown in Figure 3, the comparative analysis of participants’ engagement with the VEC and HEC via the UES questionnaire indicates that participants reported significantly higher focused attention ( Z=156.5,p<.001 ) and perceived usability ( Z=32,p<.001 ) with the VEC compared to the HEC.

However, there were no statistically significant differences between VEC and HEC in terms of esthetic appeal ( Z=561.5,p=.501 ) or reward ( Z=565,p=.47 ). A correlation analysis revealed that focused attention was positively correlated with perceived usability ( r=0.47,p<.001 ) and esthetic appeal was positively correlated with reward ( r=0.3,p=.017 ). Interestingly, the correlation between UES dimensions was different between the VEC and HEC. Specifically, perceived usability was not significantly correlated with focused attention ( r=0.05,p=.079 ) but a significant positive correlation between esthetic appeal and reward was still observed ( r=0.42,p=.016 ). With respect to participants’ ratings of the HEC, neither perceived usability and focused attention nor esthetic appeal and reward were significantly correlated. However, there was a significant negative correlation between focused attention and esthetic appeal ( r=0.43,p=.014 ). These quantitative insights into participants’ engagement with the VEC provide context for our qualitative findings on their perceptions, which are summarized in Table 3.

**Table 3.** Summary of the themes and sub-themes identified during the qualitative analysis.

Themes and sub-themes	Summary
VEC usability perception	VEC perceived as engaging due to its usability, convenience, and real-time feedback, which made exercise easier and more focused. However, system latency and voice recognition issues were noted as drawbacks.
VEC as a “Training Buddy”	Participants preferred VEC over HEC for its consistent attention, reliability, and ability to act as a training companion. While VEC alleviated social anxieties, its mechanical voice reduced engagement.
Designing for enhanced engagement and personalization Designing VEC personality to enhance engagement Fostering companionship by embodying presence Integrating motivational cues Support personalized interaction	To improve VEC, participants suggested incorporating human-like personality, embodying presence, providing motivational cues, and supporting personalization features to enhance engagement and foster a “Training Buddy” experience.

### VEC usability perception

In general, participants in this study reported having engaging experiences with the VEC largely due to its usability—a recurring theme in our interviews and supported by our quantitative results showing higher UES scores for the VEC compared to the HEC.

Participants indicated that the VEC was easy to use and intuitive, important factors for utilizing the system alone for at-home training. P11 shared their process for facilitating a quick and easy launch: “I’m trying to minimize all the steps okay between waking up and getting started.” Participants also expressed interest in continuing to use the VEC long-term, which is an indicator of user’s potential adherence to training which is important for them to achieve the benefits of regular exercise: “I think the virtual exercise coach would help me stick to my program” [P23].

Convenience was reported as another key advantage of the VEC and recognized it as something that would enable them to exercise when they have the time and space to do so: “in an everyday scenario, I think it would be more convenient with a virtual coach …being able to do it whenever wherever” [P11]. In particular, participants found the VEC useful for counting dumbbell curl repetitions, eliminating the need to track reps themselves: “You don’t have to think about what rep you’re on, just like, ’Oh, seven, cool”’ [P3]. Such feedback helped decrease cognitive load during exercise and focus on their movements: “It really encouraged me to do the exercise. Having to count myself puts a much higher mental load, whereas somebody else counting lets me focus on the physical aspect” [P14]. P23 agreed: “I could think about using a virtual coach because I get lost in the counting. Having a coach count sets for me lets me focus on the movement” [P23].

Participants perceived the VEC’s real-time feedback more accurate and objective than HEC, a perception known as machine heuristic,^69^ cultivating a sense of accountability within them during the exercise: “when I’m interacting with the person. I can just say that I’m tired and not doing it anymore. So it’s more compelling when you are interacting with the system” [P16]. Few, however, favored the HEC for their agility or adaptability: “personal trainer can coach and correct problems or can adapt” [P3].

Participants also noted the VEC’s limitations in real-time feedback, citing concerns about its responsiveness due to the system latency with respect to being ready to receive a response. The system’s latency made VEC feel less human-like, reducing its appeal: “If I was using it personally, I would. I would like if the system respond, you know, was ready for my response a little quicker. Okay. More like a human interaction” [P9]. While humans can also experience a delay in processing information from the moment of hearing a response, the system was found to be quite uncanny and thus rather a nuisance: “then I have to wait for the voice command saying ‘ready’. I feel a little bit annoyed about it maybe” [P30]. Additionally, when the VEC misunderstood what a participant said, it would prompt the participant with feedback acknowledging the system’s inability to comprehend, causing the participant to waste time and energy waiting even longer to try again: “it had some voice recognition because it’s, it made a sound when I picked it up (the weight), and then it was like, Oh, I didn’t get that and I had to wait until I could actually answer the ready question which meant more time in the actually holding the weight” [P2].

Nevertheless, VEC’s simplistic design was praised: “I would like it, like as simple as possible, actually. I think it was perfect …” [P23] and they highlighted VEC’s potential to function as a social companion in exercising, similar to having a training buddy as discussed next.

### VEC as a “Training Buddy”

Aligning with our quantitative findings on the focused attention dimension of the UES questionnaire, most participants preferred VEC over HEC for its unwavering attention and reliability during exercise session. Recalling a previous negative experience with a human coach who did not pay full attention during training, P16 stated, “I would prefer virtual coach because I know the system would be paying attention to me all the time.”

Participants discussed the VEC’s potential social and pragmatic characteristics of VEC that could enable it to serve not just as a coach, but also as a training companion, fostering emotional connections and potentially improving engagement and motivation. Many participants described being absorbed in the VEC experience and perceived it as a “training buddy,” a theme that appeared consistently in the interviews. “…if you train alone at the gym, there’s no one to help push you further. There’s no one to say. one more rep or two more reps. You’ve got it. So it’s nice that the system can push you a little bit” [P9].

Participants noted that the VEC could help alleviate the emotional insecurities associated with exercising in public, such as anxiety and lowered self-esteem that are detrimental to training motivation: “[A] lot of people exercise because of a bad self-esteem and do not have to be watched …you take the human away so you don’t feel watched” [P1]. This sentiment was echoed by P10 and P6 who mentioned the pressure and judgment that can come from being observed by others during exercise: “people have like, judgmental kind of things. They’re like, come on …You may feel like oh, he’s judging me” [P10] and “Because at home you don’t need someone else, and you have the system and no one will see how you’re struggling” [P6]. Furthermore, participants said that having an unfamiliar human coach coming into one’s house is uncomfortable and they would rather prefer a VEC in such scenario: “in a home setting, the virtual exercise coach would be more appealing because, I mean, having a human coach come into my house and stuff …” [P30].

Participants emphasized the importance of VEC’s verbal interaction qualities in user engagement. They found the VEC’s voice too mechanical, leading to disengagement: “the reason I’m not also that being that engaged, it’s the kind of mechanical way of saying things” [P13]. “I guess I just zoned out, but maybe I don’t know if it was because he was a robot. A virtual voice ” [P18]. Despite its negative impact of attention and engagement, the robotic voice was perceived as satisfactory in assistive contexts, such as shorter, simpler phrases or counting: “[Robotic] voice is fine because it’s counting a simple number” [P14].

The current version of the VEC lacks the verbal characteristics (e.g. tone and intonation) found in human coaches. Participants expressed that a human-like personality of VEC might afford users to foster a bond with the system enabling more opportunities for levity during interactions with the trainee: “I’ve had some coaches before that were like because you become friends with them. They can be like, ‘Oh, lol, that’s not a rep. You’re cheating’ …‘that doesn’t count’ to be like jokey with it” [P2]. Participants offered valuable suggestions for enhancing the VEC by incorporating more human-like interactions to make the future design of the system more engaging and user-friendly, which we discuss next.

### Designing for enhanced engagement and personalization

To improve the future design of the VEC, participants suggested enhancing engagement and personalization to foster companionship and reduce cognitive load during exercise, thus creating an emotionally engaging “Training Buddy.” They recommended designing more human-like personality in its feedback, embodying presence, providing motivational, and supporting personalized interaction within the system.

#### Designing VEC personality to enhance engagement

Participants found the content of verbal feedback and interactions with the VEC engaging: “the fact that she (VEC) was supporting and saying ‘good job’ was a relief …the fact that she was applauding me was nice” [P10]. However, they often described its voice as too mechanical or robotic, leading to disengagement during exercise sessions. As P13 noted, “the reason I’m not also that being that engaged, it’s the kind of mechanical way of saying things.” A comment from P18 supports this notion: “I guess I just zoned out, but maybe I don’t know if it was because he was a robot. A virtual voice.” Participants indicated the lack of human element in the voice degraded their VEC experience, placing it in the uncanny valley: “the human element of it, it’s hard to quantify right, but someone giving you the right tone. That was well off [with the VEC]” [P30]. Additionally, the lack of any emotional tone in VEC’s feedback made it difficult for participants to feel moved: “it was engaging but it was quite monotone didn’t have that much like emotions” [P20]. P3 highlighted the effect of the monotonous vocal tone on their physical sensation while exercising: “it [VEC’s voice] was just plain. It’s says, you can do this. But you don’t really feel it in your body.” Participants, however, did find the robotic voice acceptable in assistive contexts, such as shorter, simpler phrases or counting: “[Robotic] voice is fine because it’s counting a simple number” [P14].

Participants suggested that incorporating a more human-like personality into the system’s feedback could enhance engagement and foster a stronger bond with users, creating more opportunities for levity during interactions. P2 proposed, “I’ve had some coaches before that were like because you become friends with them. They can be like, ‘Oh, lol, that’s not a rep. You’re cheating’ …‘that doesn’t count’ to be like jokey with it.” To address the VEC’s lack of emotional expression, P20 recommended that the voice could be more enthusiastic: “saying the words with a bit more enthusiasm.” Participants also suggested incorporating warmth and motivation into the voice and noted that a more human-like quality would improve the user experience: “Being more human-like would give me a better experience with this system.”

#### Fostering companionship by embodying presence

Training with a human coach requires the trainee to be present with the coach, either virtually or physically, which often boosts motivation through a sense of presence. Several participants discussed their appreciation for being in the presence of someone, whether is the human coach or friends. For them, exercise was a social activity: “When I do physical training in the gym, I do it with my friend or friends. So we like motivating each other” [P20]. Furthermore, the shear sense of presence of even a stranger can be motivating enough to keep trainees from quitting and potentially perform better:“can’t really give up because there is someone watching me. Who, I don’t know but I have to like perform better because I just can’t give up now” [P4]. However, some participants recognized the lack of physical presence as a significant disadvantage for the VEC, “I would have a physical human being as a coach because then it would encourage me to perform more, push myself more” [P1].

Participants discussed ways to enhance the VEC’s presence and conversational interactions through embodiment, proposing ideas such as integrating animated characters: “it could just be that you could see someone (VEC) talking, …it could also be like animation” [P17]. Others expressed that mere presence of an embodied VEC may not be sufficient; rather, establishing a human-human like connection with the VEC during training sessions could significantly impact motivation. For example, P17 compared the VEC’s feedback to that of a stranger, implying that it might not be as motivating as feedback from a familiar human companion during training: “if you’re in the gym with friends, you know that your friend is standing around trying to get used to it. But when the system did it, it’s a little bit like when it’s a stranger doing it. Then it’s maybe not that encouraging.”

#### Integrating motivational cues

While participants appreciated the VEC’s real-time feedback on their dumbbell curl repetitions, they suggested that the system could be improved by providing more comprehensive updates on overall exercise progress: “it’s useful for you to keep track of how much further. When you’re getting at least halfway done, say, ’halfway done’, or ’just two more reps left’ as an additional indication, you’re almost done. ’Just keep pushing it’ and ’just keep doing it’ would have been nice” [P14].

Participants expressed believed that such feedback could reduce cognitive load and boost motivation to complete the set. They proposed that the VEC could further alleviate mental load and provide encouragement by playing music via the same speakers in which the VEC communicates, “when you go to the gym you have the music or something like it keeps you distracted …And then it’s easier to do the exercises when you are distracted” [P6].

#### Support personalized interaction

Participants offered diverse perspectives on enabling users to personalize the system in relation to improving the VEC. During interviews, many participants emphasized the importance of incorporating performance analytics to monitor their progress over time: “The app could show you your progress, like what you did last week, yesterday and today …I think it can be motivating to look back and see how, how much how far you’ve come” [P9]. Designing the system to track the user’s progress can also afford personalized recommendation for adjusting training sessions to individual users. Participants offered insights into different kind of recommendations the system could provide. P9 suggested that weights could be adjusted based on user movement analysis: “analyze the movement, analyze the weight, how easy it was and maybe tell you, it’s time to increase in weight or decrease if it’s too heavy. That would be cool” [P9]. P6, on the other hand, recommended accounting for a more holistic set of factors: “you have a like a standard weight and then the system will calculate from your muscle activity and body weight and so on from your data, …and it will adjust the weight” [P6].

## Discussion

Our findings highlight users’ positive experiences with the VEC and outline key design requirements for future voice-enabled VEC, as suggested by participants. In this section, we discuss potential challenges and their implications for designing more engaging VEC, summarized in Table 4.

**Table 4.** Challenges and implications for designing virtual exercise coach (VEC).

Challenges	Design implications
Challenge of uncanny disposition	Use VEC’s voice as a design element to create a personality that fosters a ‘Training Buddy’ experience, mimicking a human trainer
Challenge of social dynamics and presence	Incorporate embodiement visually immersive experience and to imbue social interaction.
Challenge of real time responsiveness	Improve response time and speech recognition to support more engaging human-to-human-like interaction.
Challenge of tailoring to individuals	Personalize exercise sessions according to individual user’s needs.

### Challenge of uncanny disposition

Participants in this study compared their experience with the VEC to their own prior experiences exercising with a human coach and perceived it as easy to use and offering a level of convenience unavailable with human coaches. However, VEC’s personality characteristics reflected by its verbal feedback was described as having an uncanny quality. This perceived uncanniness, attributed to the VEC’s lack of human-like vocal tone and warmth, may hinder its potential for long-term use as VEC’s mechanical and robotic voice can be detrimental to engagement and motivation. Our findings align with previous research criticizing conversational agents for their monotonous and robotic voices, suggesting the need for a more human-like voice to engage users.^18,70^

Future work should investigate how VEC personality can be designed with more human-like qualities to elicit a greater emotional connection to foster relationships between the system and users. Designing the VEC’s personality to be capable of emotional expression through verbal human-like qualities (e.g. warmth, tone, pitch, tempo, and speech patterns) could help mitigate the uncanny feeling alluded to by many of the participants and potentially trigger beneficial brain activity. While it is uncertain at this time how different users might react to the different variations of verbal qualities, our findings suggest that a more human-like personality would support a stronger emotional connection between the VEC and users that could influence the VEC’s ability to foster a bond as a “Training Buddy.”

### Challenge of integrating social dynamics and presence

Participants expressing a strong preference for working out socially—whether in group settings with friends or under the guidance of a human coach—highlights a key design challenge of incorporating social elements into VEC to mirror the engaging and motivating dynamics of human interactions during exercise. The VEC used in this exploratory study, with its scripted dialogue and lack of physical embodiment, fell short of replicating the dynamic and socially rich environment provided by a human coach. Despite these limitations, participants reported significantly higher perceived usability and focused attention with the VEC, suggesting they found it to be an acceptable substitute for human presence. This is further supported by the lack of significant differences in participants’ responses to the Reward and Aesthetic dimensions between the VEC and HEC, as reported through the UES questionnaire (see Figure 3). A possible explanation for this outcome is that the HEC was instructed to maintain neutrality, avoiding emotional expressions and using the same script as the VEC. These instructions nullified the advantages of dynamic interactions and non-verbal communication, which humans naturally engage in. As a result, participants described HEC they interacted with outside the study context as friendly, using levity to enhance exercise sessions and build rapport.

However, as prior work postulates, how users perceive embodiment is affected by the concept of proxemics—users’ perception of embodiment based on the proximity of objects—and as such, should be considered in future designs of the VEC.^71^ For example, in a gym setting, a HEC would typically stand within a social proximity bubble, fostering a sense of connection. In contrast, during remote exercise sessions, the perceived distance of the HEC, determined by factors such as camera zoom, influences how trainees interpret emotions and interactions.

Looking ahead, the future design of VECs would benefit from embodiment to offer a more visually immersive interaction experience, facilitating more human-like interactions. Enhancing the presence of VEC with human-like qualities could increase the potential for forming a “Training Buddy” like relationship between the user and the system.^18,70^ Careful considerations on how to establish a sense of presence through embodiment can help foster a bond between the user and the VEC, positioning it as a companion rather than a mere tool.

### Challenge of responsiveness

Participants in this study often experienced frustration with the VEC due to its slow response times and poor speech recognition. These issues led to noticeable delays, resulting in complaints about wasted energy and the need for participants to repeat themselves or speak louder. In contrast, the HEC was perceived as more responsive, agile, and intuitive, effectively handling various speech patterns and vocal characteristics with minimal delay. This disparity highlights a critical challenge of improving response time and speech recognition to enhance user experience and sustain engagement.

Difficulties in speech recognition may also increase when a user is tired and not able to speak as clearly during intense workouts or have disabilities that affect normal verbal interactions. If these issues in speech timing and recognition become too frequent, users might find the interactions feel unnatural, which can negatively impact VEC’s usability and user engagement.^72^ Future VEC should focus on improving response time and speech recognition to realize the potential benefits of VEC in engaging users to exercise, as emphasized by Paay et al.^53^

### Challenge of tailoring to individuals

Our study reveals that participants desire a system capable of customizing feedback and exercise sessions according to their individual performance and lifestyle, including specific recommendations on exercises, weight amounts, and guidance to enhance exercise precision. For example, one participant described how the VEC could balance hand strength by customizing weights to strengthen a weaker hand, bringing it up to the strength of the other hand.

Experienced HEC have the advantage of intuition and experience, enabling them to make recommendations based on their observations, such as adjusting weights when a trainee struggles. This intuitive approach allows HEC to provide tailored advice without needing prior data about the trainee. In contrast, a data-driven VEC can use collected information to create more efficient and effective exercise routines that leverage users’ strengths and address their weaknesses. Additionally, participants expressed a desire to track their progress throughout their exercise journey. This historical data could also help the VEC offer recommendations for training schedules and preferences.

Our findings align with previous research^73^ which highlights the positive impact of personalized coaching on user engagement and motivation in the context of physical activity. Prior work has pointed to a number of promising strategies, including goal setting, user targeting, adaptation, and context awareness.^44,74^ Further work is needed to elucidate the effects of these personalization mechanisms on long-term engagement with VEC.

### Limitations

The generalizability of these results is subject to a number of limitations. Firstly, the VEC used text-to-speech and speech-to-text API’s from Microsoft Cognitive Services for the verbal interaction and feedback. We observed sizable lag, and the difficulties in understanding the participants’ input causing the participants to become irritated when interacting with the system. These issues with lag and responsiveness are common challenges faced by researchers in the voice user interface.^72^ The system design also ran into issues when giving feedback and simultaneously needing to begin counting again which resulted delay counting sometimes causing confusion to participants. It would have been better for the system to count internally and pick up again after the next movement following the completed delivery of the feedback.

Secondly, this study was conducted in a controlled laboratory setting with a small number of participants, each engaging in only a single VEC session lasting no more than an hour. Consequently, the generalizability of our results to users’ daily lives may be limited. Therefore, a longitudinal study involving a larger and more diverse participant pool is necessary to gain a deeper understanding of users’ perceptions of the system. These sessions should also be conducted outside the lab to assess the VEC in real-world scenarios.

Finally, the exoskeleton prototype used in our research was constructed using LEGOs, which served well for rapid prototyping but lacked durability and the ability to easily adjust for different arm sizes. This lack of adjustability resulted in the prototype breaking partially on several occasions. Future studies should consider employing a more robust exoskeleton.

### Future work

Recent development of large language model (LLM) like ChatGPT that enable more natural verbal interactions between the VEC and users can address the challenges of uncannyness and response lag we found in this study. Future research could explore integrating such LLM-powered conversational agents with VEC and assess the impact of verbal communication on user engagement and motivation. Future studies could also explore physical or virtual embodiments of the VEC and explore its impact on user experience. Enhancing an agent’s ability to express emotions could lead to deeper interactions and longer engagement. Over time, a consistent pattern of emotional expressions might contribute to the agent developing a perceived personality, fostering a stronger connection with the user.

Research could explore the development of intelligent VEC capable of tailoring exercise sessions by adapting to users’ physical abilities and using real-time and historical performance data. These systems could, for instance, adjust the difficulty level of workouts and offer personalized training recommendations to better meet individual needs. Participants in this study identified this features as essential for enhancing the value of VEC over HEC, given the VEC’s ability to offer objective assessments.

Future exoskeleton-based VEC could physically assist users in tasks like lifting weights. Such assistance hold significant potential for physical rehabilitation, especially in motivating stroke patients during therapy sessions. Studies suggest that lack of motivation is one of the main barriers to stroke rehabilitation.^75–77^ Although strategies like serious games have been studied and proven effective for patient engagement,^78–80^ exploring alternative means such as verbal feedback can be valuable to address diverse motivational needs of patients. A recent study of 362 rehabilitation professionals identified verbal interactions such as active listening, praise, and enjoyable communication as top strategies to motivate stroke patients for rehabilitation.^81^ Future VEC designs could incorporate these elements to provide the encouragement patients need to stay committed to their rehabilitation.

## Conclusion

The widespread inactivity of the world’s adult population highlight the pressing need for more effective strategies to engage people in regular exercise. In this context, enhancing user engagement through VEC that offer a human-coach-like experience is crucial for increasing exercise participation and helping individuals achieve its physical and psychological benefits. In response to this challenge, this study developed a voice-based VEC, compared user engagement with the VEC and HEC, and gathered user perceptions and recommendations to guide the future design of the such VEC technology. Findings suggest a strong potential of the technology in providing engaging exercise experience and that VEC could benefit from adopting a more human-like personality to foster emotional connections. Enhancing the system’s human-likeness could involve creating a sense of presence by embodying the VEC and improving response time during exercise. Based on these insights, we have identified key design challenges and implications for developing VEC that support engaging physical exercises. Future development should focus on integrating LLM and incorporating features to adapt to the users’ needs, such as those of stroke patients during their rehabilitation.

## Notes

a. Otter is an AI-powered transcription software. https://otter.ai/.
